# Patent ductus arteriosus in a lamb: A case report

**Published:** 2016-03-15

**Authors:** Afshin Jafari Dehkordi, Farzaneh Hoseini

**Affiliations:** *Department of Clinical Sciences, Faculty of Veterinary Medicine, Shahrekord University, Shahrekord, Iran.*

**Keywords:** Echocardiography, Lamb, Necropsy, Patent ductus arteriosus

## Abstract

Patent ductus arteriosus (PDA) is a persistent patency of a vessel normally present in the fetus that connects the pulmonary arterial system to the aorta. The ductus arteriosus fails to close at birth when breathing commences and placental blood circulation is removed. Closure of the ductus arteriosus arises in response to decline pulmonary vascular resistance and increased systemic vascular resistance. This report describes a case of PDA in a two-month-old male lamb with clinical signs of machinery murmur, tachycardia, increase respiratory rate, weakness and ill thrift. Echocardiographic examination and necropsy finding confirmed PDA.

## Introduction

 There are some clinical reports of congenital cardio-vascular defects in veterinary literature.^   1 ^ The cause of congenital cardiac abnormalities is unknown. However, it is supposed that they result from injury during development or from single recessive genes or polygenic sets that have lesion-specific effects on cardiac development.^2^^,^^3^ 

Patent ductus arteriosus (PDA) is a persistent patency of a vessel (normally present in the fetus) that connects the pulmonary arterial system to the aorta. The ductus arteriosus fails to close at birth when breathing commences and placental blood circulation is removed. Closure of the ductus arteriosus arises in response to decline pulmonary vascular resistance and increased systemic vascular resistance.^   4 ^

The PDA is rare as a single defect in large animals. It can occur with other cardiac anomalies. In large animals the most common other defects reported with PDA are tetralogy and pentalogy of Fallot and pseudo truncus arteriosus.^   2 ^

Normal foals may have a PDA for a few days after birth. However, closure of the ductus arteriosus is expected by 96 hr of age. Normal ruminants rarely have a PDA after birth, and if one is present it is considered abnormal. Functional closure may precede anatomic closure of the PDA. Diagnosis of this defect is uncommon in older animals. Currently there is no evidence to suggest that this is an inherited defect in horses or cattle.^   2 ^

## Case description

A 2-month-old male lamb with a history of weakness and ill thrift was examined in the Veterinary Clinic of Shahrekord University, Iran. Clinical examination revealed high pitch character murmur that was auscultated on the left and right cardiac area. This murmur waxed and waned during cardiac auscultation (machinery murmur). Therefore, presence of PDA was suspected based on occurrence of machinery murmur. Also, there was tachycardia, increase respiratory rate and normal body temperature. No cyanotic mucus membranes and no significant hematological abnormalities were observed.

**Fig. 1:**
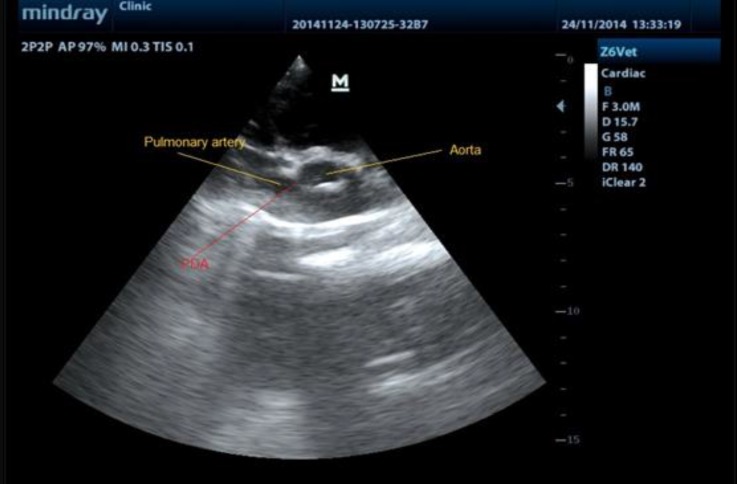
Echocardiogram showing patent ductus arteriosus

**Fig. 2.**
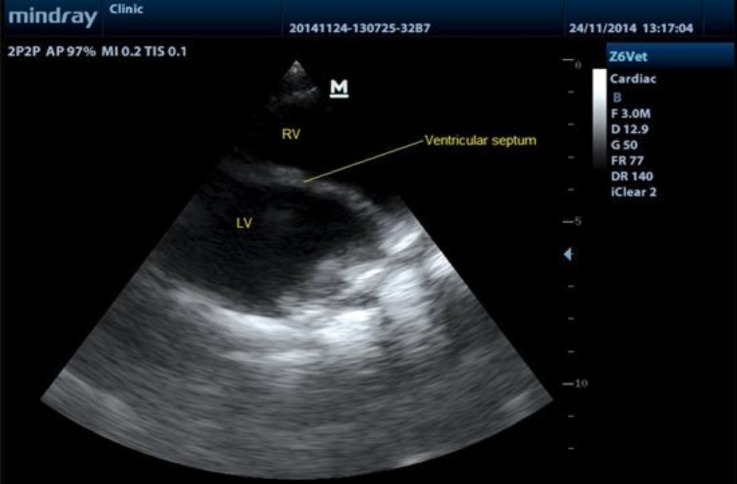
Echocardiogram showing normal ventricular septum

Echocardiography was performed from right para-sternal long axis view by an ultrasonography device (Model Z6 Vet; Mindray Medical Ltd., Shenzen, China). Linear phased array probe with 3 MHz frequency was used. Ultrasonographic waves were sent to the base of heart which aorta, pulmonary artery and a shunt between them was seen clearly (Fig. 1). In addition, there was not any abnormities and shunts in the ventricular and atrial septum that ventricular septal defect (VSD) and atrial septal defect (ASD) were not observed in ultrasonography (Fig. 2).

**Fig. 3:**
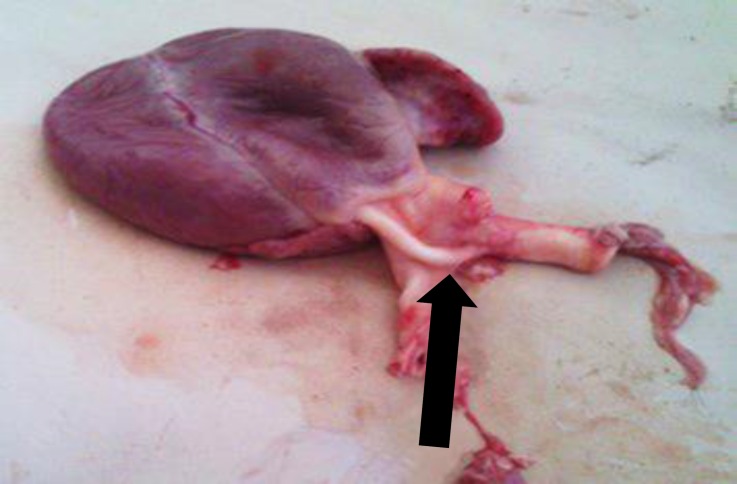
Patent ductus arteriosus (black arrow) with a diameter of 0.25 cm and 1.10 cm in length adjoining the descending aorta and pulmonary artery

The lamb was slaughtered due to poor prognosis. On postmortem examination, gross lesions were limited to the heart. There was a shunt between pulmonary artery and aorta (Fig. 3).

Therefore, Echocardiographic findings and postmortem examination confirmed the diagnosis of PDA in this lamb.

## Discussion

The ductus arteriosus is a large channel found normally in all mammalian fetuses that allows most of the blood from the right ventricle to bypass the fetus's fluid-filled non- functioning lungs. Normally the ductus arteriosus narrows near term and tightens rapidly after birth in response to lowered pulmonary vascular resistance, increased systemic vascular resistance, increased blood volume, and increased left ventricular pressure when breathing begins and the placental blood circulation is removed. If the ductus arteriosus is large or the resistance to flow across the ductus is minimal, there is a significant left-to-right shunt which generates a large left ventricular volume overload. The left ventricular response may be failure or, with time, development of dilation (primarily) and hypertrophy. These processes will result in pulmonary hypertension and congestion. The right ventricle can be affected by the pulmonary pressure load, and right ventricular hyper-trophy can also develop. If the pulmonary resistance equals or exceeds the systemic vascular resistance, a right-to-left shunt happens.^1^^,^^2^ 

The ductus arteriosus can be of various length and diameter, however, is patent between the aorta and the pulmonary artery. The PDA often enters the aorta caudal to the origin of the brachiocephalic trunk. Changes in the left and right ventricles and lung and pulmonary vasculature are variable and related to the size of the shunt.^   5 ^

When the PDA is large, there may be cardiomegaly with left atrial and left ventricular dilation, right ventricular hypertrophy, pulmonary congestion, and edema.^4^^,^^6^ 

The clinical signs of PDA depend on the length and diameter of the ductus arteriosus, direction of the shunted blood, and presence of other cardiac anomalies.^1^^,^^2^  

The PDA produces a loud continued murmur associated with the left-to-right shunting of blood from the aorta to the pulmonary artery. The intensity of murmur waxes and wanes with each cycle because of the effects of normal pressure changes the blood flow giving rise to name of machinery murmur. The systolic part of murmur is very loud and usually audible in most of the cardiac auscultatory area but the diastolic component is much softer and limited to the base of the heart. The intensity of the murmur gains with increased heart rate, exercise, or excitement. The pulse is large in amplitude, however, has a low diastolic pressure.^1^^,^^2^ 

Sporadically the PDA is manifested by a holosystolic murmur because the diastolic component is barely audible, except for the left heart base. Large PDAs can exist without producing a murmur. In the animal with augmented pulmonary vascular resistance and reversal of the shunt, there may be cyanosis of the caudal parts of the body if the PDA enters the aorta caudal to the brachio-cephalic trunk.^1^^,^^2^^,^^6^

A PDA should be suspected when a continuous, high-pitched murmur, frequently referred to as a "machinery murmur" because of its alternating intensity, is auscultated. The murmur may be auscultated on the left and right sides of the thorax, however, is usually loudest in the left third or fourth intercostal space at the level of the shoulder.^1^^,^^2^ 

No characteristic clinicopathologic changes are related to a PDA. Radiography may show enlargement of the cardiac silhouette and pulmonary over-circulation in an uncomplicated PDA. Pulmonary venous congestion, interstitial pulmonary edema, and alveolar edema are evidences of a large PDA with left-sided heart failure. These signs are not specific for PDA and can be present with any congenital cardiac anomaly that result in a left-to-right shunt. No consistent electrocardiographic pattern has been identified with PDA. Echocardiographic evidence of a PDA is provided by the finding of an enlarged left atrium and left ventricle with a pattern of left ventricular volume overload and increased values for the ratio of the left atrial to aortic root dimension. Direct visualization of the ductus arteriosus is difficult with echocardiography but is most successful when performed from the left cardiac window.  ^2^^,^^4^^,^^7^^,^^8^ 

A PDA arising from the pulmonary artery has been imaged echocardiographically in an 11-month-old Friesian- Holstein heifer. High-velocity turbulent flow throughout the cardiac cycle in the pulmonary artery and ductus arteriosus is detected with pulsed wave, continuous wave, or color flow Doppler echocardiography.^   2 ^

Cardiac angiocardiography and nuclear angio-cardiography using a selective aortic angiogram provide final evidence of a PDA. Oximetric data show a step-up in oxygen content or saturation in the pulmonary artery that is proportional to the size of the shunt. Indicator dilution methods also provide evidence of a left-to-right shunt occurring in the pulmonary artery in cases of an un-complicated PDA. Pulmonary arterial and right ventricular pressures may be elevated with a large PDA.^   2 ^ The PDA is a common cardiovascular abnormality in dogs but infrequent in other species.^9^  Wiseman and Murray reported the first case of uncomplicated PDA in the bovine.^   10 ^

Sandusky and Smith described two cases of PDA in a series of 1000 autopsies on calves and cattle,^   11 ^ while Gopal and others in a 14-year study of calves with cardiac defects, declared five PDA out of 36 calves with cardiac defects.^   12 ^
